# Differential expression of genes related to gain and intake in the liver of beef cattle

**DOI:** 10.1186/s13104-016-2345-3

**Published:** 2017-01-03

**Authors:** C. M. Zarek, A. K. Lindholm-Perry, L. A. Kuehn, H. C. Freetly

**Affiliations:** USDA, ARS, U.S. Meat Animal Research Center, Clay Center, NE 68933-0166 USA

**Keywords:** Feed efficiency, Transcriptome, Steers, Beef cattle, Immune response, Metabolism, Cellular transport

## Abstract

**Background:**

To better understand which genes play a role in cattle feed intake and gain, we evaluated differential expression of genes related to gain and intake in the liver of crossbred beef steers. Based on past transcriptomics studies on cattle liver, we hypothesized that genes related to metabolism regulation and the inflammatory response would be differentially expressed. This study used 16 animals with diverse gain and intake phenotypes to compare transcript abundance after a 78 day ad libitum feed study.

**Results:**

A total of 729 genes were differentially expressed. These genes were analyzed for over-representation among biological and cellular functions, and pathways. Cell transport processes and metabolic processes, as well as functions related to transport, were identified. Pathways related to immune function, such as the proteasome ubiquitination pathway and the chemokine signaling pathway, were also identified.

**Conclusions:**

Our results were consistent with past transcriptomics studies that have found immune and transport processes play a role in feed efficiency. Gain and intake are impacted by complex processes in the liver, which include cellular transport, metabolism regulation, and immune function.

**Electronic supplementary material:**

The online version of this article (doi:10.1186/s13104-016-2345-3) contains supplementary material, which is available to authorized users.

## Background

The highest production cost for beef cattle is feed, so it is important to select cattle that can produce more meat, while consuming less feed [1]. Thus, an understanding of differentially expressed genes and over-represented gene clusters and pathways in organs like the liver that may impact gain and feed intake in beef cattle, is critical. In addition, these genes and pathways may result in biological markers for the identification or selection of animals with superior phenotypes.

The liver was chosen because of its role in metabolism. Gluconeogenesis, glycolysis, and glyconeogenesis regulate blood glucose levels and occur to a large extent in the liver. The phosphogluconate oxidative pathway accounts for a significant portion of glucose oxidation in the liver [2]. In ruminant digestion, the microorganisms in the rumen break down plant material to the volatile fatty acids acetic acid, propionic acid, and butyric acid, which are absorbed through the rumen [3]. The liver produces approximately 85% of the glucose turnover in ruminants, which is a key process for energy production in ruminants [4]. Huntington [5] states that the liver metabolizes 50–90% of the butyrate and propionate absorbed by the rumen. The liver is also a major organ for glycogenesis, storage of glycogen, and glycogenolysis, which also help to regulate the blood glucose level. In addition, fatty acids are removed from the blood to provide energy via beta-oxidation. Another function of the liver is amino acid metabolism. Amino acids are removed from the bloodstream and used for protein synthesis, metabolism or catabolism [6]. Other functions of the liver include synthesis of albumin and fibrinogen, detoxification of blood and waste removal, and conversion of ammonia absorbed from the gut and rumen into urea [6]. In addition, the liver, portal veins, spleen, and gut use about one-half of the total heat energy in cattle, which is far greater than what would be predicted based on the mass of organs [5].

Studies analyzing the liver’s role in feed efficiency using residual feed intake (RFI) have been conducted previously on cattle. Weber et al. [7] found differentially expressed genes involved in lipid metabolism and inflammation were down-regulated in the livers of low RFI Angus bull progeny. In 2011 Chen [8] found that low RFI bulls tended to have an up-regulation of genes related to the extracellular matrix and a down-regulation of genes involved in xenobiotic metabolism. From these findings, they concluded that the more efficient animals are better at moving substrates across their membranes and that the less efficient animals have higher levels of cellular stress in the liver [8]. Alexandre et al. [9] showed increased oxidative stress and hepatic lesions in the liver of low feed efficiency Nelore bulls. They suggested the altered lipid metabolism causes more reactive oxygen species, leading to inflammation. The inflammation compromises liver tissue in the low efficiency bulls, allowing easier entry for pathogens and increasing the immune response that was not seen in the high efficiency bulls [9].

Rather than RFI, this study uses high and low gain, and high and low feed intake quadrants as phenotypes to evaluate differences in transcript abundance in the liver. These data may help explain the molecular pathways that are important for efficient gain and reduced intake in the liver.

## Methods

### Animal care and use

The U.S. Meat Animal Research Center (USMARC) Animal Care and Use Committee reviewed and approved all animal procedures. The procedures for handling cattle complied with the Guide for the Care and Use of Agricultural Animals in Agricultural Research and Teaching (FASS [10]).

### Feed trial

**Fig. 1:**
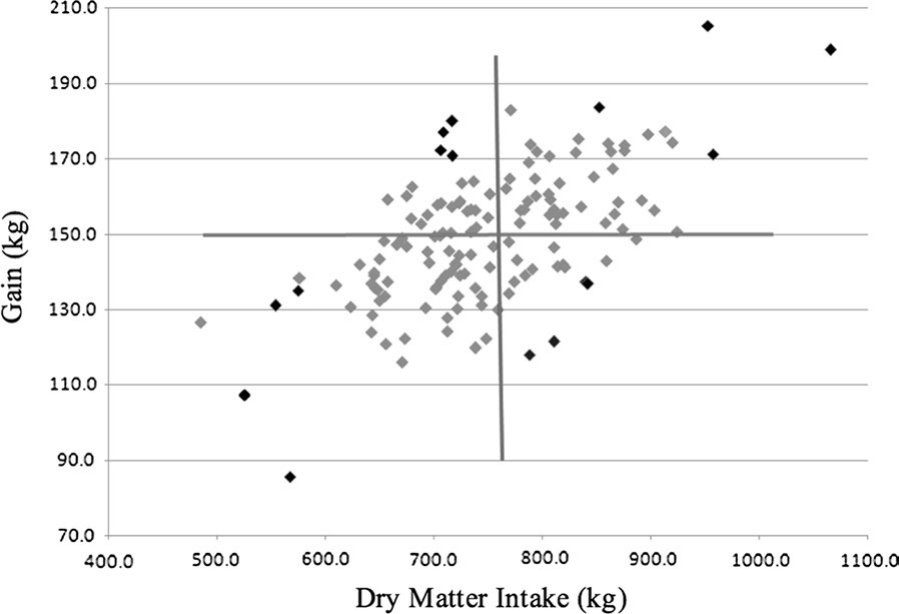
Total dry matter intake and total gain for steers on feed trial (n = 143). The average for gain is represented by the *horizontal line* and the average for intake is represented by the *vertical line*. The bivariate mean is the intersection of these lines. Steers chosen for the study are represented by the *black data* points

One hundred forty-three 11-month-old crossbred steers were fed ad libitum for 78 days. The finishing diet consisted of dry-rolled corn (57.35%), ground alfalfa hay (8%), Steakmaker with Tylan (4.25%), urea (0.4%), and wet distillers grain with solubles (30%). Daily intake of the steers was measured using the Insentec feeding system (Marknesse, The Netherlands), and steers were weighed every 3 weeks to record gain. Steers were also weighed at the beginning of the feed trial on day 0 and day 1, and on the last 2 days of the feed trial. Sixteen steers were selected for harvest based on maximizing their distance from the bivariate mean of total gain and intake in four quadrants: high gain–high intake; high gain–low intake; low gain–low intake; and low gain–high intake were selected from the study (Fig. 1). The medical records and representation of breed within phenotypic groups were also considered while selecting steers for harvest, such that sire breed was not over-represented within groups (Table 1). Animals selected for the experiment were euthanized by captive bolt followed by exsanguination. Tissues were collected from steers harvested over 4 days, with one steer from each phenotypic group harvested each day to reduce environmental effects between groups. A sample of the right lobe of the liver was diced and flash frozen in liquid nitrogen. The samples were stored at −80 °C until RNA isolation.

**Table 1:** Cattle breeds within each phenotypic group

Breed	High gain–high intake	High gain–low intake	Low gain–low intake	Low gain–high intake
Angus	0.25	0.25	0.008	0.125
Brahman	0	0	0	0.125
Brangus	0	0.031	0	0
Braunvieh	0	0	0.047	0
Brown Swiss	0	0	0.188	0
Charolais	0.25	0.25	0.254	0.25
Gelbvieh	0.125	0.242	0.004	0.063
Hereford	0	0.063	0.254	0
MarcII^a^	0	0.008	0	0
MarcIII^b^	0	0.016	0.004	0.063
Red Angus	0	0	0.086	0
Santa Gertrudis	0	0	0	0.188
Shorthorn	0	0.078	0	0
Simmental	0.359	0	0.156	0.188
Unknown^c^	0.016	0.063	0	0

^a^MarcII animals are 0.25 Simmental, 0.25 Gelbvieh, 0.25 Hereford, and 0.25 Angus

^b^MarcIII animals are 0.25 Pinzgauer, 0.25 Red Poll, 0.25 Hereford, and 0.25 Angus

^c^Unknown animals have breed of unknown origin

### RNA Isolation

Hepatic RNA was isolated according to the manufacturer’s protocol for Trizol (Ambion, Carlsbad, CA, USA), with a few exceptions. Briefly, a six-station Omni Prep homogenizer (Omni International, Kennesaw, GA, USA) was used to homogenize 50–100 mg of liver in 1 mL of Trizol for 40 s. The first centrifugation was lengthened to 20 min (at 11,750 rcf) from the standard 15 min, and the pelleted RNA was washed twice with 70% ethanol. The RNA pellets were suspended in 50–150 µL of nuclease-free water, depending on the size of the pellet. Genomic DNA was removed from the extracted RNA using the Qiagen RNeasy mini-kit (Valenci, CA, USA) with a gDNA eliminator column, according to the manufacturer’s protocol. A Nanodrop 8000 spectrophotometer (Thermo Scientific, Wilmington, DE, USA) was used to measure the concentration of the total RNA. The average 260/280 ratio was 1.93 (range 1.91–2.15). The RNA integrity number was measured using an Agilent Bioanalyzer and a RNA 6000 Nano kit (Santa Clara, CA, USA). The range of the RNA integrity numbers (RIN) was 4.9–8.7, with an average of 6.7. All samples had a RIN of 6.0 or higher, except two samples, which had RINs of 4.9 and 5.9.

### Microarray

The samples were prepared according to the Affymetrix GeneAtlas System (Santa Clara, CA) microarray protocol. Briefly, 250 ng of RNA and *Poly*-*A* RNA controls were reverse-transcribed to cDNA. The cDNA was transcribed to cRNA, which was purified with Agencourt AMPure XP beads (Beckman Coulter Life Sciences, Indianapolis, IN, USA) and washed with 80% ethanol. Fifteen microgram of cRNA was then reverse-transcribed to single-stranded cDNA. RNase was added to the ss-cDNA to remove the remaining RNA, and then the ss-cDNA was purified with AMPure beads (Indianapolis, IN) and washed with 80% ethanol. 5.5 μg of ss-cDNA was fragmented and biotinylated with the WT Expression Kit. The labeled ss-cDNA, *bioB*, *bioC*, *bioD*, *cre* controls, and Control Oligonucleotide B2 were hybridized to Bovine 1.1ST array strips (Santa Clara, CA) for 20 h at 48 °C. The array strips were then stained and washed by the GenetAtlas Fluidics station and Wash and Stain Kit for WT Array Strips (Santa Clara, CA) according to protocol and imaged by the Affymetrix GeneAtlas scanner. CEL files produced by the scanner were normalized and converted to CHIP files using the Affymetrix Expression Console software. The Guanine Cytosine Count Normalization and Signal Space Transformation algorithms and Robust Multichip Analysis are conducted by the software as part of the normalization and processing. The GCC normalizes the intensities of the signals read by the scanner. The cel and chp files can be accessed at the Gene Expression Omnibus (GEO) with accession number GSE75700. Differential expression between the phenotypic groups described above was determined by one-way ANOVAs for each Cartesian quadrant (3° of freedom). *P* values were multiple-test corrected for false discovery rate using the Benjamini and Hochberg calculations [11].

### Gene function and pathway analysis

Differentially expressed genes were analyzed with the Protein Analysis Through Evolutionary Relationships (PANTHER) statistical over-representation test for *Bos taurus* with the default parameters, except the Bonferroni correction for multiple testing was not used. The over-represented GO-Slim biological processes and molecular functions are summarized in Tables 2 and 3. PANTHER Version 10.0, released May 15, 2015 was used in the analysis [12, 13].

**Table 2:** Over-represented biological processes identified by PANTHER

Biological process	GO annotation	Number of genes	Expected number of genes	Nominal P value
Transport	GO:0006810	61	48.86	4.06E−02
Ion transport	GO:0006811	24	14.13	9.09E−03
Cation transport	GO:0006812	22	11.39	2.93E−03
Carbohydrate metabolic process	GO:0005975	20	10.94	7.92E−03
Mitosis	GO:0007067	13	6.79	2.09E−02
Polysaccharide metabolic process	GO:0005976	11	3.5	9.62E−04
Nucleobase-containing compound transport	GO:0015931	7	2.27	8.56E−03
Sensory perception	GO:0007600	5	12.14	1.74E−02

**Table 3:** Over-represented molecular functions identified by PANTHER analysis

Molecular function	GO annotation	Number of genes	Expected number of genes	Nominal P value
Transporter activity	GO:0005215	37	21.81	1.36E−03
Transmembrane transporter activity	GO:0022857	34	19.81	1.76E−03
Cation transmembrane transporter activity	GO:0008324	17	7.46	1.65E−03
Cytoskeletal protein binding	GO:0008092	10	4.78	2.36E−02
Amino acid transmembrane transporter activity	GO:0015171	6	1.7	7.91E−03
Intermediate filament binding	GO:0019215	2	0.22	2.01E−02

Ingenuity® Pathway Analysis (IPA®) by Qiagen was also used for differential expression analysis to identify genes over-represented in canonical pathways. The top five pathways with the lowest P value (*P* < 0.05) were reported by the system.

### Validation

The RNA was reverse-transcribed to cDNA using iScript™ Reverse Transcription Supermix for qRT-PCR (BioRad, Hercules, CA, USA) according to the manufacturer’s protocol. Briefly, RNA was read using a Nanodrop 8000 spectrophotometer (Thermo Scientific, Wilmington, DE) and diluted to 1 μg in 16 μL of water. The iScript (4 μL) was added and placed on a Dyad Peltier Thermal Cycler (BioRad, Hercules, CA, USA) at 25 °C for 5 min, 42 °C for 30 min, and 85 °C for 5 min. The cDNA was used for validating the microarray by qRT-PCR. Primers were designed using Primer3 [14, 15]. USB VeriQuest® SYBR Green qPCR Master Mix (2×) with Fluorescein (Affymetrix, Santa Clara, CA, USA) was used with 10 μM primers according to protocol. The gene *RPLPO* (P value 0.4211 for gain and 0.7179 for intake) was used as a housekeeper, and the results were normalized to its expression. The polymerase chain reactions (PCR) were run on a C1000 Touch Thermal Cycler, CFX384 Real-Time System (BioRad, Hercules, CA, USA) at 95 °C for 10 min, followed by 40 cycles of 95 °C for 15 s, and 60 °C for 30 s. The 2^−ΔΔCt^ was calculated according to Livak and Schmittgen [16] then log transformed for data analysis. Validation assays were analyzed with the same 3° of freedom Cartesian quadrant model. Genes selected for validation, oligonucleotide primers, and amplicon sizes are listed in Additional file 1: Table S1.

## Results

Analysis of microarray data identified 729 differentially expressed genes (nominal *P* ≤ 0.05; Additional file 2: Table S2). Of these, 449 genes were annotated. None of the differentially expressed genes passed a false discovery rate correction for multiple testing. To determine whether genes were over-represented among pathways or by gene function, they were analyzed using PANTHER and IPA.

The PANTHER over-representation test identified biological processes related to cellular transport and metabolism (Table 2). Five transport processes and two metabolic processes were identified. The top three biological processes with the greatest number of differentially expressed (DE) genes were transport processes, two of which were related to ion transport. Another 20 genes were classified as carbohydrate metabolism and 13 DE genes were identified as belonging to the process of mitosis, which had a fold change of 1.91.

The PANTHER analysis also identified six molecular functions that were over-represented by the DE genes (Table 3). Four of the six were a form of transporter activity, with two focused on transmembrane transportation. The other two molecular functions also have cellular transportation implications since they were cytoskeletal protein binding and intermediate filament binding. The cytoskeleton helps to move vesicles, molecules, and organelles within cells, which underscores the transportation function identified by PANTHER.

Several IPA pathways showed that the differentially expressed genes are related to differences in immune responses. The protein ubiquitination pathway, selenocysteine biosynthesis II pathway, and chemokine signaling pathway were identified by IPA (Table 4). Fifteen DE genes were in the proteasome ubiquitination pathway, two in the selenocysteine biosynthesis II pathway, and five in the chemokine signaling pathway. The genes in each pathway are listed in Table 5.

**Table 4:** Top canonical pathways identified by IPA analysis for DE genes

Pathway	Number of DE genes	Percentage of genes (%)^a^	Nominal P value
Protein ubiquitination pathway	15	6.4	2.42E−03
Selenocysteine biosynthesis II (Archaea and Eukaryotes)	2	33.3	1.07E−02
Chemokine signaling	5	8.1	2.86E−02

^a^The percent of DE genes in the pathway in relation to the total number of genes in that pathway is given

**Table 5:** DE genes identified by IPA

Pathway	Gene symbol	Gene name	Nominal P value
Proteasome ubiquitination	*DNAJC22*	DnaJ heat shock protein family (Hsp40) Member C22	5.12E−03
	*DNAJC5*	DnaJ heat shock protein family (Hsp40) Member C5	2.06E−02
	*DNAJB2*	DnaJ heat shock protein family (Hsp40) Member B2	3.50E−02
	*DNAJC16*	DnaJ heat shock protein family (Hsp40) Member C16	3.78E−02
	*TAP2*	Transporter associated with antigen processing 2	5.39E−03
	*USP40*	Ubiquitin specific peptidase 40	9.94E−03
	*USP26*	Ubiquitin specific peptidase 26	4.55E−02
	*UCHL1*	Ubiquitin C-terminal hydrolase L1	3.40E−02
	*SKP1*	S-phase kinase-associated protein 1	1.39E−02
	*USP14*	Ubiquitin specific peptidase 14	3.97E−02
	*USP44*	Ubiquitin specific peptidase 44	2.82E−02
	*PSMC5*	Proteasome 26S subunit, ATPase 5	4.77E−02
	*PSMA5*	Proteasome subunit alpha 5	3.74E−02
	*PSMD5*	Proteasome 26S subunit, non-ATPase 5	3.11E−02
	*SMURF1*	SMAD specific E3 ubiquitin protein ligase 1	3.28E−02
Chemokine signaling	*PTK2B*	Protein tyrosine kinase 2 beta	2.72E−02
	*CAMK2B*	Calcium/calmodulin dependent protein kinase ii beta	3.28E−02
	*CAMK4*	Calcium/calmodulin-dependent protein kinase IV	3.74E−02
	*PLCG1*	Phospholipase C, gamma 1	3.83E−02
	*KRAS*	Kirsten rat sarcoma viral oncogene homolog	3.86E−02
Selenocysteine biosynthesis II	*PSTK*	Phosphoseryl-TRNA kinase	4.30E−02
	*SEPSECS*	Sep (O-phosphoserine) TRNA:Sec (Selenocysteine) TRNA synthase	3.01E−02

Validation of differentially expressed genes was performed using qRT-PCR for twelve genes. Three of the genes were significantly associated with the phenotypes tested (*P* < 0.1), and 7 of the 12 genes showed qRT-PCR expression patterns consistent with the microarray data (Table 6).

**Table 6:** Microarray and qRT-PCR expression of genes chosen for validation

	qRT-PCR				Microarray			
	Group X^a^	Group Y^a^	Fold change^b^	Nominal P value	Group X^a^	Group Y^a^	Fold change^b^	Nominal P value
*AGPAT3*	0.039	0.099	1.15	0.53	10.63	11.38	1.68	0.03
*DNAJB2*	0.094	−0.099	−1.56	0.64	8.8	8.22	−1.49	0.04
*FABP3*	−0.32	−0.019	2.00	0.008	6.75	7.47	1.65	0.02
*IGFBP1*	0.12	−0.37	−3.09	0.29	15.48	11.84	−12.47	0.009
*NAT1*	−0.49	−0.31	1.51	0.54	10.02	10.74	1.65	0.03
*NCAPG*	0.67	0.26	−2.57	0.59	6.21	5.77	−1.36	0.008
*PLK3*	−0.18	−0.02	1.45	0.06	7.02	7.41	1.31	0.03
*PPP1CA*	−0.021	0.1	1.32	0.37	10.19	10.54	1.27	0.05
*PTK2B*	0.054	−0.16	−1.64	0.18	7.05	7.67	1.54	0.03
*SMAD6*	0.16	0.11	−1.12	0.15	7.13	7.74	1.53	0.001
*TAP2*	0.12	0.26	1.38	0.39	9.11	10.02	1.88	0.005
*TIGAR*	−0.18	0.09	1.86	0.09	7.54	7	−1.45	0.02

^a^For *AGPAT3*, *DNAJB2, SMAD6,* and *TAP2,* Group X was high gain-low intake and Group Y was low gain-high intake. *FABP3, PTK2B, PLK3*, and *TIGAR* had high gain-high intake (Group X) and low gain-high intake (Group Y). *IGFPB1, NAT1,* and *NCAPG* had high gain-low intake (Group X) and low gain-low intake (Group Y). *PPP1CA* had high gain-high intake (Group X) and low gain-low intake (Group Y)

^b^Expression of Groups X and Y (the groups with the greatest differential expression) were compared to determine fold change for each gene

## Discussion

The purpose of this study was to determine whether DE genes in the liver contribute to variation in gain and feed intake in beef steers. In total, 729 genes were identified as differentially expressed in the liver. Although the individual genes identified as differentially expressed did not pass FDR, they were over-represented among pathways and gene functions. Moreover, the pathways and processes found by analyzing our DE genes are similar to other transcriptomic studies, suggesting that these results have biological relevance. It is likely that these genes did not pass FDR due to a small sample size (n = 16) of 4 animals/phenotypic group or due to the large number of breeds represented in the study. These data could be used for further analysis with additional data from other tissue types from these same animals or as part of a meta-analysis with similar data from other populations of cattle.

Similar “transport” processes and functions were identified as over-represented among the differentially expressed genes by PANTHER for both biological and molecular function tests suggesting that transportation or movement of substances within or among cells is associated with gain and intake in the liver of beef cattle. This is supported by the RFI study conducted by Chen et al. [8] with Angus bulls. They found an up-regulation of extracellular matrix genes and genes involved in cell movement and organization in the low RFI bulls. Chen et al. [8] suggest that the up-regulation of genes involved in transportation suggests greater cellular proliferation and growth in the low RFI animals, compared to the high RFI animals. Similar gene families, such as solute carrier family genes and collagen genes, were identified by both Chen et al. [8] and this study. The pathways identified by IPA were representative of immune response, suggesting that cellular stress may have impacted the growth of the steers. For example, the proteasome ubiquitination pathway marks proteins for degradation by binding them to ubiquitin, and has been shown to be important for cellular growth, as well as immune function. The proteasome removes damaged proteins, and cleaves some proteins to active forms that may influence transcriptional regulation or other processes, such as inflammation. Further, the proteasome cleaves proteins for MHC-I antigen presentation and degrades some antigens during an immune response [17]. The *TAP2* codes for a transmembrane transporter located in the endoplasmic reticulum that functions with *TAP1* to move MHC I peptides into the ER. It is up-regulated by IFN-beta, TNF-alpha, and IFN-gamma [18]. In this study, *TAP2* was up-regulated among the animals with high intake. We postulated that the high gain–high intake animals were under less oxidative stress, which seems to be supported by the differential expression of components of the protein ubiquitin pathway in this study. This suggests that more efficient animals expend less energy degrading cellular components, and thus are able to utilize that energy for gain.

The selenocysteine biosynthesis pathway and chemokine signaling pathways also imply that the steers’ immune system may impact their gain and intake. Selenocysteine is an amino acid that is incorporated into several proteins that require selenium to function. Some of these proteins play a role in reducing oxidative stress, while others are required for selenium transport or synthesis of other compounds [19]. Chemokines are signaling molecules that recruit leukocytes to sites of infection or injury by binding to G-protein coupled receptors on the plasma membrane and initiating a cascade. These cascades can lead to transcriptional regulation, chemotaxis, or cell polarization, which prepares the cell for movement [20]. Both of these pathways have components related to immune function and were shown to be differentially expressed between the gain and intake phenotypes.

Some of the genes in the chemokine signaling pathway have been found to have metabolic roles in addition to their immunological roles. For example, *CAMK2* is needed for glucose production, and is activated by glucagon in the liver. Further, it was shown to activate the transcription factor *FOXO*-*1*, which activates transcription of the genes that lead to glucose production (glucose-6-phosphatase and phosphoenolpyruvate carboxykinase). The *CAMK2* may also play a role in obesity through its regulation of glucose production in the liver [21]. The *CAMK2* expression in this study was highest in the high gain–low intake animals.

The inclusion of distiller’s grains with solubles resulted in a diet that exceeded the minimum protein level recommended for growing cattle. Since the liver is the primary site of catabolism of excess amino acids, and is the primary site of ureagenesis, elevated protein potentially could result in an increase in expression of genes associated with those metabolic processes. However, we did not detect genes involved in ureagenesis as differentially expressed between animals with high and low intake in this study.

Other transcriptome studies of cattle liver have found immune response to play a role in feed efficiency. Connor et al. [22] conducted a feed restriction study followed by ad libitum feeding study with black angus steers to study differential expression in the liver. Genes involved in cell proliferation were lower in transcript abundance during the restriction period, and cytokinesis, inflammation, and oxidative stress were higher in transcript abundance after the ad libitum feeding [22]. Paradis et al. [23] used RNA sequencing to identify differentially expressed genes in liver biopsies from beef replacement heifers with low or high RFI adjusted to backfat thickness. They found seven DE genes, five of which are regulated by interferon signaling. They also used qRT-PCR to identify DE genes and identified genes that play a role in oxidative stress and detoxification. These results showed that more efficient heifers may have an improved response to inflammation and may expend less energy combating pathogens than high RFI heifers [23]. Chen et al. [8] saw a down-regulation of genes involved in inflammation and oxidative stress in high feed efficiency Angus bulls, and an up-regulation of those genes in the low efficiency bulls [8].

## Conclusions

In conclusion, this study utilized microarray to identify processes that may impact gain and feed intake in beef cattle. While our results did not pass multiple testing correction, they suggested that immune response and oxidative stress play a role in feed efficiency. The proteasome ubiquitination pathway and chemokine pathways were over-represented by our DE genes, underscoring potential immune functions that underlie some of the variation in gain and intake.

## Additional files

**Additional file 1: Table S1.** Forward and reverse primers for qRT-PCR validation. Sequences of oligonucleotides designed for qRT-PCR validation of differentially expressed genes. Table also includes GenBank ID used for primer design, length of primers and expected amplicon size (bp).

**Additional file 2: Table S2.** Differentially expressed genes in steer liver. LSMEANS by phenotypic quadrant, nominal P values and FDR p values are presented. Table includes the list of differentially expressed genes with nominal P < 0.05.

## Abbreviations

DE: differentially expressed
ER: endoplasmic reticulum
FDR: false discovery rate
IPA: ingenuity pathway analysis
qRT-PCR: quantitative real time polymerase chain reaction
PANTHER: Protein Analysis through Evolutionary Relationships
PCR: polymerase chain reaction
RFI: residual feed intake
RIN: RNA integrity number
