# Supplementary material for: Differential expression of genes related to gain and intake in the liver of beef cattle
Source: BMC Res Notes. 2017 Jan 3;10:1. doi: 10.1186/s13104-016-2345-3 (PMC5217266; doi:10.1186/s13104-016-2345-3)
Supplement: Supplementary file 1 — Additional file 1: Table S1. Forward and reverse primers for qRT-PCR validation. Sequences of oligonucleotides designed for qRT-PCR validation of differentially expressed genes. Table also includes GenBank ID used for primer design, length of primers and expected amplicon size (bp). [file 13104_2016_2345_MOESM1_ESM.docx]

| Supplementary Table 1. | | | | | |
| --- | --- | --- | --- | --- | --- |
| Forward and reverse primers for qRT-PCR validation. | | | | | |
| Gene | Name | RefSeq number | Primer Sequence 5'-3' | Primer length | Product length |
| *AGPAT3* | 1-acyl-sn-glycerol-3-phosphate acyltransferase gamma | NM_001038046 | gacttcctctgtgggtggac | 20 | 96 |
|  |  |  | gatgaggggcacgtagagc | 19 |  |
| *DNAJB2* | dnaJ homolog subfamily B member 2 | NM_001034592 | acctctatggccgggaag | 18 | 90 |
|  |  |  | aggtgaaggtgaagccaggt | 20 |  |
| *FABP3* | fatty acid binding protein 3 | NM_174313.2 | ctcttctgccagtgggctac | 20 | 101 |
|  |  |  | caggagaatttccccagtca | 20 |  |
| *IGFBP1* | insulin-like growth factor-binding protein 1 precursor | NM_174554 | gagagcttgagggctcttga | 20 | 96 |
|  |  |  | ctccctggctaatctgtcca | 20 |  |
| *NAT1* | arylamine N-acetyltransferase 1 | NM_001075572 | ttggaaaagaccctgatgct | 20 | 99 |
|  |  |  | ttgtccatcgcattgatgat | 20 |  |
| *NCAPG* | non-SMC condensin I complex subunit G | NM_001102376.2 | aggatacaggcagttcttgctc | 22 | 127 |
|  |  |  | ataacactgcccgcctaactt | 21 |  |
| *PLK3* | serine/threonine-protein kinase PLK3 | NM_001075153 | actatgtggccccagaagtg | 20 | 102 |
|  |  |  | ggggactcccacatagcag | 19 |  |
| *PPP1CA* | serine/threonine-protein phosphatase PP1-alpha catalytic subunit | NM_001035316 | gcatgacctggacctcatct | 20 | 105 |
|  |  |  | cagtagttgggggctgaaaa | 20 |  |
| *PTK2B* | protein-tyrosine kinase 2-beta | NM_001102252 | actgtggacctggtcattgg | 20 | 109 |
|  |  |  | ggaggcatctgatggacttg | 20 |  |
| *SMAD6* | mothers against decapentaplegic homolog 6 | NM_001206145 | caccccatcttcgtcaactc | 20 | 103 |
|  |  |  | cgaagtcgaacaccttgatg | 20 |  |
| *TAP2* | antigen peptide transporter 2 | NM_174222 | tattttggccgtgtgattga | 20 | 106 |
|  |  |  | cacacagagagctcccaaca | 20 |  |
| *TIGAR* | fructose-2,6-bisphosphatase TIGAR | NM_001076370 | gcatgggattttggagaaga | 20 | 92 |
|  |  |  | cttctgcaaccccatatttc | 20 |  |
